# Fingerprint Recognition with Identical Twin Fingerprints

**DOI:** 10.1371/journal.pone.0035704

**Published:** 2012-04-27

**Authors:** Xunqiang Tao, Xinjian Chen, Xin Yang, Jie Tian

**Affiliations:** 1 Center for Biometrics and Security Research, Institute of Automation, Chinese Academy of Sciences, Beijing, China; 2 Department of Electrical and Computer Engineering, University of Iowa, Iowa City, Iowa, United States of America; 3 Life Science Research Center, School of Electronic Engineering, Xidian University, Xi'an, Shaanxi, China; Institute of Psychology, Chinese Academy of Sciences, China

## Abstract

Fingerprint recognition with identical twins is a challenging task due to the closest genetics-based relationship existing in the identical twins. Several pioneers have analyzed the similarity between twins' fingerprints. In this work we continue to investigate the topic of the similarity of identical twin fingerprints. Our study was tested based on a large identical twin fingerprint database that contains 83 twin pairs, 4 fingers per individual and six impressions per finger: 3984 (83*2*4*6) images. Compared to the previous work, our contributions are summarized as follows: (1) Two state-of-the-art fingerprint identification methods: P071 and VeriFinger 6.1 were used, rather than one fingerprint identification method in previous studies. (2) Six impressions per finger were captured, rather than just one impression, which makes the genuine distribution of matching scores more realistic. (3) A larger sample (83 pairs) was collected. (4) A novel statistical analysis, which aims at showing the probability distribution of the fingerprint types for the corresponding fingers of identical twins which have same fingerprint type, has been conducted. (5) A novel analysis, which aims at showing which finger from identical twins has higher probability of having same fingerprint type, has been conducted. Our results showed that: (a) A state-of-the-art automatic fingerprint verification system can distinguish identical twins without drastic degradation in performance. (b) The chance that the fingerprints have the same type from identical twins is 0.7440, comparing to 0.3215 from non-identical twins. (c) For the corresponding fingers of identical twins which have same fingerprint type, the probability distribution of five major fingerprint types is similar to the probability distribution for all the fingers' fingerprint type. (d) For each of four fingers of identical twins, the probability of having same fingerprint type is similar.

## Introduction

Biometrics refers to the automatic identification of a person based on his or her physiological or behavioral characteristics. These methods have advantages over traditional token based identification approaches using a physical key or access card, and over knowledge based identification approaches that use a password for various reasons. First, the person to be identified is required to be physically present at the point of identification to provide his or her biometric traits. Second, identification based on biometric characteristics avoids the need to carry a card or remember a password. Finally, the biometric characteristics of identified person cannot be lost or forged. During the past few decades, a number of verification systems based on different biometric characteristics have been proposed [1]. Any physical or behavioral characteristics which can be used as verification to recognize a person must satisfy the following requirements 0: (i) universality (everyone possesses the characteristic); (ii) permanence (the characteristic remains invariant over a life time); (iii) collectability (the characteristic is easy to capture); and (iv) distinctiveness (the characteristic is different for everyone). The performance of biometric verification systems highly depends on the distinctiveness of the biometric characteristics. However, not all biometrics provide sufficient information to verify different people, especially identical twins.

**Figure 1:**
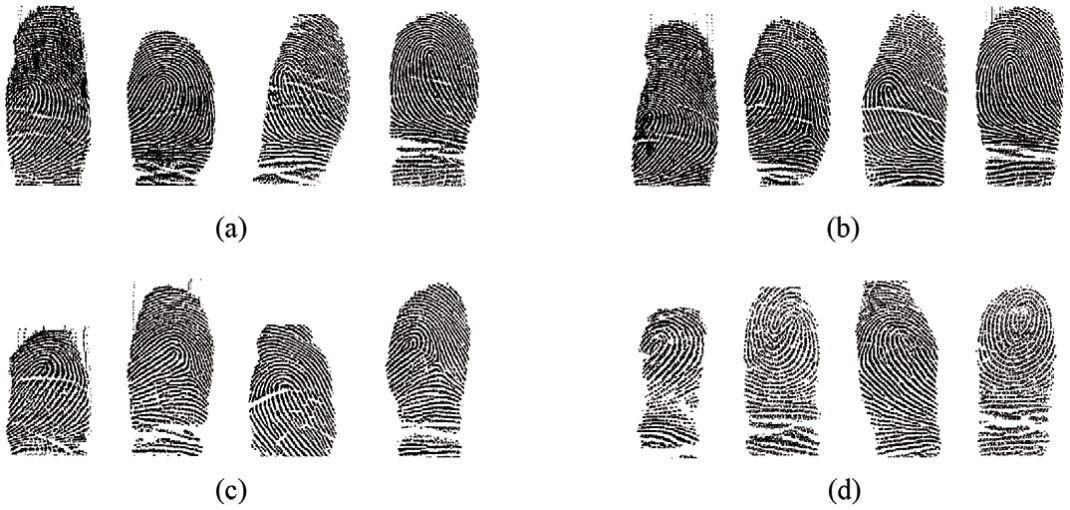
Some examples of fingerprint images in our database. (a) are fingerprint images of four fingers of the first twin, and (b) are the fingerprint images of the corresponding four fingers of his/her identical twin. (c) and (d) show fingerprint images from a non-identical twin pair.

**Figure 2:**
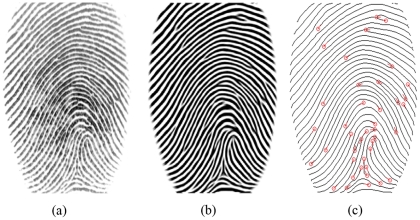
An example of fingerprint enhancement and minutiae extraction by P071 method. (a) Original fingerprint; (b) Enhancement results of (a); (c) Minutiae extraction results of (a).

There are two basic types of twins: dizygotic, commonly referred to as fraternal twins and monozygotic, referred to as identical twins [2]. Dizygotic twins result from two eggs that are fertilized separately by two different sperms. This usually happens when the mother produces more than one egg at ovulation. The two fertilized eggs develop separately and have their own genes. They may or may not be the same gender. Monozygotic twins result from one fertilized egg. This egg divides into two individuals who will share all of their genes in common. These twins are genetically identical, with the same chromosomes and similar physical characteristics and, therefore, they cannot be distinguished using the same deoxyribonucleic acid. The frequency of identical twins is about 0.4% across different populations [3]. Some researchers believe that this is the performance limit of face recognition system [4]. Hence, studying the ability of biometric traits to discriminate between identical twins is an important issue for biometric verification. As the biometrics-based verification becomes more pervasive, there have existed some studies (e.g. Fingerprint [2] [5]–[7], Palmprint [8], Speaker [9], and Iris [10]) which determined the distinctiveness of the biometric characteristics in order to establish the performance limits of such systems. Jain et al. 0 analyzed the similarity between twins' fingerprints in a study using fingerprint images from 94 pairs of identical twins. They obtained the twin-twin imposter distribution of matching scores computed by matching a fingerprint with his/her identical twin sibling (twin-twin match) and twin-nontwin imposter distribution of matching scores between a person's fingerprint and everyone else except his/her twin (twin-nontwin match). Then, they obtained genuine distribution of the matching scores from a standard public domain fingerprint database (e.g. NIST9 CD No.1). The experimental results showed that the fingerprint verification systems could be used to distinguish identical twins. In another analysis of fingerprints from 66 pairs of twins 0, Han et al. also found that fingerprints can be used to identify identical twins with an insignificant drop in the performance: the Equal Error Rate (EER) generally increased by 1–2% compared to nontwin impostor matching. Srihari et al. [6] analyzed the similarity between twins' fingerprints in a study using fingerprint images from 298 pairs of twins. The authors analyzed this similarity using two-level features. With the features of level 1, they found that twins' fingers are much more likely (55%) to have the same pattern type than non-twins' fingers (32%). With the features of level 2, they concluded that the similarity between the fingerprints from twin fingers is higher than from two arbitrary fingers. Sun et al. [7] analyzed the similarity between twins based on multiple biometric traits (fingerprint, face, and iris). For the fingerprint identification, the used database is only a part of the database used in this paper. Kong et al. [8] used 1028 palmprint images from 53 pairs of identical twins' palms. They made two different twin matches. In the first experiment, they matched the palmprints from the pairs of identical twins' palms (called real twin match). In the second experiment, they matched the left and right palmprints from the same person (called virtual twin match). The authors found that palmprints generated from the same genetic information were significantly correlated; however, they still could be distinguished with non-genetically related information. Ariyaeeinia et al. [9] presented investigations based on a speech database from 49 pairs of identical twins. The authors performed two verification tests: OVERALL and TWIN tests. In the OVERALL tests, any speaker could claim the identity of any other speaker in the registered population. In the TWIN tests, each registered speaker could only claim the identity of him/herself or that of his/her own identical twin. The Equal Error Rate reported was 1.0% for the TWIN tests and 0.5% for the OVERALL tests. The first patent for iris recognition asserted that twins irises were different:“Not only are the irises of the eyes of identical twins different, but the iris of each eye of any person is different from that of his other eye” [11].

**Figure 3:**
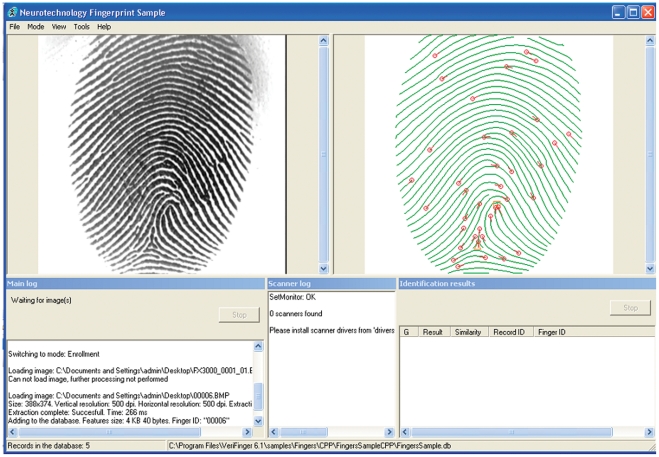
An example of fingerprint enhancement and minutiae extraction with user interface of VeriFinger 6.1.

This paper presents the continued investigations of the ability of fingerprint verification technology to distinguish between identical twins. The investigations are tested on a large identical twin fingerprint database which contains 83 twin pairs, 4 fingers per individual and six impressions per finger: 3984 (83*2*4*6) images. Comparing to the pioneers' work [2] [5] [7], our contributions are as follows:

1. Compared to all the methods [2] [5] [7], two state-of-the-art fingerprint identification methods: P071 and VeriFinger 6.1 are used for twin fingerprint identification in this paper rather than one fingerprint identification method in [2] [5] [7].
2. Compared to Jain's [2] and Srihari's [6] methods, six impressions per finger were captured rather than just one impression, which makes the genuine distribution of matching scores more realistic. As we know, the genuine distribution of matching scores needs to be estimated from matching multiple fingerprint impressions of the same finger. In both Jain's and Srihari's databases, due to only a single impression for each finger was captured, the distribution of the genuine scores has to be synthesized, i.e., it is not from the real genuine matching.
3. Compared to Sun et al.'s method [7], the fingerprint database is from the same source. However, only a part of the fingerprint dataset (51 pairs) was used in [7], while the whole fingerprint dataset (83 pairs) is used in this paper.
4. A novel statistical analysis is conducted for five major fingerprint types, which aims at showing the probability distribution of the fingerprint types for the corresponding fingers of identical twins which have same fingerprint type. This is novel in our paper.
5. A probability analysis is conducted for four fingers from identical-twins, which aims at showing which finger has higher probability of having same fingerprint type. This is also novel in our paper.

The rest of this paper is organized as follows. Sec. 2 describes the twin fingerprint database and the state-of-the-art automatic fingerprint verification matcher for this study. Sec. 3 presents our experimental results and analysis. Sec. 4 provides a summary and conclusion.

**Figure 4:**
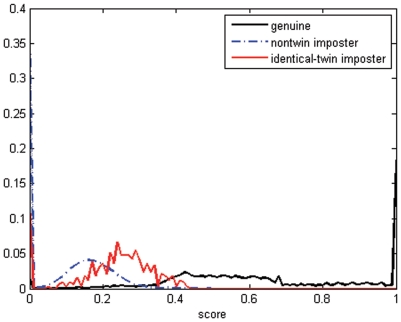
Score distributions for genuine, identical-twin imposter and non-twin imposter by P071 method.

**Figure 5:**
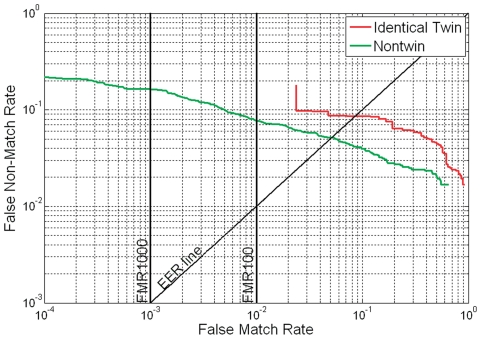
ROC curves for identical-twin and non-twin matchings by P071 method.

## Materials and Methods

### 2.1 Identical Twins Fingerprint Database

The database was collected on October 2, 2007 at the Beijing Chaoyang Park during the Fourth Beijing Twins Culture Festival. The fingerprint images were captured by a sweep sensor sw6888 0 with a resolution of 500DPI. The database includes 3984 (3984 = 83×2x×4×6) fingerprint images which from 83 pairs of identical twins, and four different fingers (left index, left middle, right index and right middle) were scanned for each person. The number of impressions for each finger was six. The finger was scanned consecutively six times to get the six impressions. All of the images were captured on the same day (single data captured session). Fig 1 shows some examples of fingerprint images in our database. Fig 1(a) shows fingerprint images of the four fingers for the first sibling of an identical twin pair, and Fig 1(b) shows the fingerprint images of the corresponding fingers for the second sibling of an identical twin pair. Figs 1(c) and (d) follow the same scheme for a non-identical twin pair. It is interesting to note that for identical twins, all four pairs of the corresponding fingers have the same pattern type; while for non-identical twins, only two pairs of corresponding fingers for non-identical twins have the same pattern type.

**Table 1:** The relationship between FAR and matching threshold.

FAR (false acceptance rate)	Matching threshold (score)
100%	0
10%	12
1%	24
0.1%	36
0.01%	48
0.001%	60
0.0001%	72
0.00001%	84
0.000001%	96

### 2.2 Identification Fingerprint Methods Overview

In this paper, two state-of-the-art methods are used to identify the similarity of twin fingerprints: P071 [13] and VeriFinger 6.1 SDK (VF6.1) 0. The details are given as follows.

**Figure 6:**
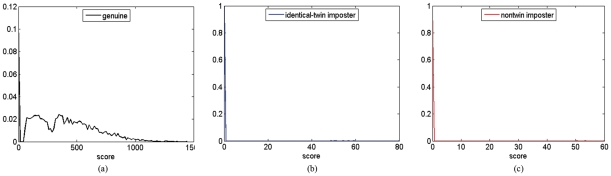
Score distributions. Score distributions for (a) genuine, (b) identical-twin imposter and (c) non-twin imposter via VeriFinger 6.1 SDK.

### 2.2.1 P071 Algorithm

P071 algorithm is first used for the identification of twin fingerprints which has been evaluated in the Fingerprint Verification Competition 2004 (FVC2004) and the performance was ranked No.3 among all of the participated algorithms. The detailed performance of the proposed algorithm on FVC2004 can be seen from the website [15]. The P071 method was based on a normalized fuzzy similarity measure. The algorithm has two main steps. First, the template and input fingerprints were aligned. In this process, the local topological structure matching was introduced to improve the robustness of the global alignment. Second, the method of normalized fuzzy similarity measure was introduced to compute the similarity between the template and input fingerprints. Two features are selected: the number of matched sample points (*n*) and the mean distance difference of the matched minutiae pairs (*d*) in the process of similarity computing. Fuzzy features were used to represent *n* and *d*. Each character is associated with a fuzzy feature that assigns a value (between 0 and 1) to each feature vector in the feature space. The value, named degree of membership, illustrates the degree of similarity of the template and input fingerprints.

Feature *n* is represented by fuzzy feature 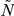 whose membership function, 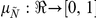, is defined as:
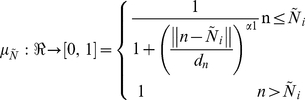
(1)Where 
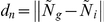
 represents the distance between 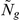 and 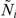. 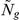 and 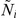 are the genuine and imposter match clusters center of fuzzy set 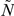.

Feature *d* is represented by fuzzy feature 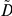 whose membership function, 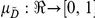, is defined as:
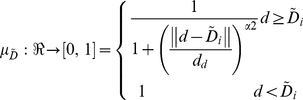
(2)Where 
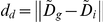
 represents the distance between 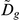 and 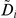. 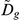
and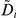 are the genuine and imposter match clusters center of fuzzy set 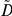.

In order to achieve the better classification performance, 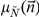 and 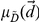 are combined. The product rule was used to compute the similarity between the template and input fingerprints as follows,

Similarity  = 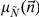
*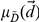 (3)

Cappelli et al. [16] have given high comments for the P071 algorithm, stating that it exhibited good tradeoffs between speed and accuracy: achieving the third-best average EER, with an average comparison time of 0.67 seconds. Fig 2 shows an original fingerprint image with its enhancement and minutiae extraction results.

**Table 2:** Performance of identical-twin and non-twin matchings using VeriFinger 6.1 SDK in terms of EER, FMR100 and FMR1000.

	EER (%)	FMR100 (%)	FMR1000 (%)
Identical-twin	5.8333	0.0000	12.2490
Non-twin	5.3843	0.0000	0.0000

**Table 3:** The probability distribution of five major fingerprint types/classes for all the fingers in our database.

Left loop(*pl*)	Right loop (*pr*)	Arch(*pa*)	Tented arch (*pt*)	Whorl(*pw*)	Total
191	185	15	7	266	664
0.2877	0.2786	0.0226	0.0105	0.4006	1.00

### 2.2.2 VeriFinger 6.1 SDK

VeriFinger 6.1 SDK [14] is a world-well-known commercialized fingerprint recognition software, which is based on an advanced fingerprint recognition technology and is intended for biometric system developers and integrators. The technology assures system performance with fast, reliable fingerprint matching in 1-to-1 and 1-to-many modes and comparison speeds of up to 40,000 fingerprints per second. VeriFinger 6.1 SDK has many features: (1) NIST MINEX proven reliability; (2) robust processing of poor quality and deformed fingerprints; (3) more than 50 scanners are supported by VeriFinger SDK. Some of the functions for VeriFinger 6.1 SDK are listed as follows:

**Table 4:** The fraction of the same fingerprint type between the corresponding fingers of identical twins.

Same	Different	All
247	85	332(83*4)
0.7440	0.2560	1.0000

**Table 5:** The probability distribution of five major fingerprint types/classes for the fingers of identical twins which have same fingerprint type.

Left loop(*pl*)	Right loop (*pr*)	Arch(*pa*)	Tented arch(*pt*)	Whorl(*pw*)	Total
73	68	5	1	100	247
0.2955	0.2753	0.0203	0.0040	0.4049	1.00

- Enroll fingerprint. Fingerprint can be enrolled from the image or by using fingerprint the scanner.
- Enroll fingerprint with generalization. Using this option, several fingerprints can be enrolled and features generalized.
- Verification. Using this option, one fingerprint can be verified against the other (1:1 matching).
- Identification. Using this option, the fingerprint is identified against an internal database (1: N matching).

**Table 6:** The fraction of the same fingerprint type for each of the four fingers of identical twins.

	Same	Different	All
**Left Index**	65(0.7831)	18(0.2169)	83(1.0000)
**Left Middle**	63(0.7590)	20(0.2410)	83(1.0000)
**Right Index**	59(0.7108)	24(0.2892)	83(1.0000)
**Right Middle**	60(0.7229)	23(0.2771)	83(1.0000)

**Table 7:** The comparing results summary of Jain, Han, Srihari, Sun and our methods.

Method	Database(pairs×members×fingers ×times)	Matcher	Matching Results(Identical-twin vs. Non-twin)	Class Correlation(Identical-twin vs. randomly chosen)
**Jain ** [**2**]	94×2×1×1 (184)	Minutiae [18]	FAR: 2-6% higher	0.7750 vs. 0.2718
**Han ** [**5**]	66×2×3×10	Minutiae [5]	EER: 1-2% higher	0.6455 vs. 0.1373Base on Tab. 2 [5]
**Srihari ** [**6**]	298×2×10×1	NFIS [19]	FPR:6.17% vs. 2.91%	0.5500 vs. 0.3200
**Sun ** [**7**]	51×2×4×2	VeriFinger [14]	EER:6.79% vs. 4.40%	NA
**Our** **Method**	83×2×4×6	P071 [13]	EER:8.67% vs.5.94%	0.7440 vs. 0.3215
		VeriFinger [14]	EER: 5.83% vs. 5.38%	

VeriFinger fingerprint recognition algorithm follows the commonly accepted fingerprint identification scheme, which uses a set of specific fingerprint points (minutiae). However, it contains many proprietary algorithmic solutions, which enhance the system performance and reliability. Some of them are listed below:

- Adaptive image filtration algorithm allows to eliminate noises, ridge ruptures and stuck ridges, and extract minutiae reliably even from poor quality fingerprints;
- VeriFinger includes a fast template matching algorithm that is tolerant to fingerprint translation, rotation and deformation.
- VeriFinger does not require the presence of the fingerprint core or delta points in the image, and can recognize a fingerprint from any part of it.

An example of fingerprint enhancement and minutiae extraction with user interface in VeriFinger 6.1 is shown in Fig 3.

### 2.3 Testing methods

To study the similarity of identical twin fingerprints, three distributions were generated: genuine distribution, identical-twin imposter distribution and non-twin imposter distribution. The genuine distribution was obtained by matching each image of one finger against the remaining images of the same finger. The identical-twin imposter distribution was obtained by matching each image of a person with his/her identical twin. The non-twin imposter distribution was obtained by matching a person with everyone else except for his/her identical twin. To sum up, a total of 9 960 (83×2×4×6×5/2) genuine matching, 332 (83*4) identical-twin imposter matching and 435 584 (83*2*82*2*16) non-twin imposter matching runs were conducted. All of the experiments were conducted on a PC Intel Core2 E6500 @ 2.33 GHZ.

Identical twins have the same chromosomes and similar physical characteristics and, therefore, they have a high class/type similarity in their fingerprints. It is well known that the class/type similarity is, to a certain extent, related to the fingerprint matching perfomance. It is our hypothesis that identical twins have higher class correlation, and the higher class correlation causes a higher similarity, i.e. it is more difficult to tell the difference between identical twins than non-identical twins. To prove our hypothesis, we manually classified 83 pairs of identical twin fingerprints in our database into five types (left loop, right loop, arch, tented arch and whorl). And the analysis for each type was then performed.

## Results

### 3.1 P071 experimental results

Fig 4 shows the genuine, identical-twin imposter and non-twin imposter distributions. The identical-twin imposter distribution was found to be shifted to the right of the non-twin imposter distribution. This indicated that identical-twin fingerprints are generally more similar than non-twin fingerprints. The same result can also be found in Fig 5 which shows the receiver operating characteristics (ROC) 0 based on Fig 4. As expected, the ability of P071 to distinguish identical twins is lower than its ability to distinguish non-twins.

### 3.2 VeriFinger 6.1 SDK experimental results

The VeriFinger 6.1 SDK matching algorithm provides a value of the features matching score as a result. The higher the score, the higher the probability that the features obtained are from the same person. The different matching threshold is linked to the different false acceptance rate (FAR). The relationships can be found in Table 1 (from VeriFinger 6.1 SDK Developer's Guide [14]). The higher the threshold, the lower the FAR and the higher the FRR (false rejection rate, the same subjects are erroneously accepted as being different) and vice versa. We obtained the matching score by the VeriFinger 6.1 SDK matching algorithm. Fig 6(a), (b) and (c) show the score histograms for the genuine distribution, identical-twin imposter distribution and non-twin imposter distribution respectively. In the genuine distribution in Fig 6(a), we can see that about 90% of the genuine matches have a score of more than 48, whereas the FAR is 0.01%. This is due to the high similarity between the two images of the same person. From Fig 6(b) and Fig 6(c), we find that about 99.99% of the non-twin imposter matches and about 99.1% of the identical twin imposter matches have a score of 0. The EER results are present in Table 2. In [5], the authors found that fingerprints can be used to identify identical twins with an insignificant drop in the performance: the Equal Error Rate (EER) generally increased by 1–2% compared to nontwin impostor matching. We also find that the automatic fingerprint verification matcher VeriFinger 6.1 SDK can distinguish between identical twins with a slightly lower accuracy than in non-twins (5.8333% vs. 5.3843%).

### 3.3 Fingerprint Class Correlation Analysis of Identical Twins

All of the fingers were manually classified into five classes (Left loop, Right loop, Arch, Tented arch and Whorl). The proportion of appearance of each of the five major fingerprint types in our database is shown in Table 3. The fraction of the same fingerprint type between the corresponding fingers of identical twins is found to be 0.7440 in our database, as shown in Table 4. Based on Table 3, if we randomly choose two fingerprint images, the probability that these two fingerprints will have the same type is equal to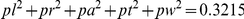, where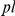,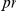,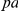,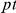 and 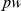 are the probabilities of a fingerprint belonging to the class/type of left loop, right loop, arch, tented arch and whorl, respectively. Thus, the probability that two randomly chosen fingers have the same class/type is only 0.3215, which is much lower than the 0.7440 that two identical twins have the same class/type. Compared to the result of the class correlation in Jain's method [2] (0.775, only the class correlation of the index fingers was analyzed in that paper), we have very similar result (0.744). However, compared to the result in Srihari's method [6] (0.55, ten fingers were used to analyze the class correlation), we have much higher probability.

A statistical analysis is conducted for five major fingerprint types/classes, which aims at showing the probability distribution of the fingerprint types for the corresponding fingers of identical twins which have same fingerprint type. To the best of our knowledge, this is the first report about it. The results are shown in Table 5. Compared to Table 3, we can find that the probability distribution is similar to the probability distribution of five major fingerprint types for all the fingers in our database.

Another probability analysis is conducted for four fingers from identical-twins, which aims at showing which finger has higher probability of having same fingerprint type. This is also novel in our paper. The results are shown in Table 6. The experiment results show that the probability of having same fingerprint type for each finger of identical twins is similar, although the left index finger has a bit higher similarity.

## Discussion

In this paper, we have investigated the ability of the fingerprint verification matcher to discriminate between identical twins. The experimental results demonstrated that the identical twins can be distinguished by a state-of-the-art method P071 and the commercial fingerprint matcher VeriFinger 6.1.

Our research is a continued investigation in [2] [5] [7]. Table 7 summarized the comparisons of Jain [2], Han [5], Srihari [6], Sun [7] and our methods. From the results, we can find that the automatic fingerprint verification system can successfully distinguish identical twins though with a slightly lower accuracy than non-twins based on no matter which identification method. All the methods (no class correlation analysis in Sun [7]) show that twins' fingers are much more likely to have the same pattern type than non-twins' fingers.

In summary, our contributions are as follows:

1. Compared to all the methods [2] [5] [7]., two state-of-the-art fingerprint identification methods: P071 and VeriFinger 6.1 are used for twin fingerprint identification in this paper rather than one fingerprint identification method in [2] [5] [7].
2. Compared to Jain's [2] and Srihari's [6] methods, six impressions per finger were captured rather than just one impression, which makes the genuine distribution of matching scores more realistic. In both Jain's and Srihari's databases, due to only a single impression for each finger was captured, the distribution of the genuine scores has to be synthesized, i.e., it is not from the real genuine matching.
3. Compared to Sun et al. 's method [7], the fingerprint database is from the same source. However, only a part of the fingerprint dataset (51 pairs) was used in [7], while the whole fingerprint dataset (83 pairs) is used in this paper.
4. A novel statistical analysis which aims at showing the probability distribution of the fingerprint types for the corresponding fingers of identical twins which have same fingerprint type has been conducted. The experimental results showed that the probability distribution of five major fingerprint types is similar to the distribution for all the fingers' fingerprint type.
5. A novel analysis which aims at showing which finger from identical twins has higher probability of having same fingerprint type has been conducted. The results show that the probability of having same fingerprint type for each finger of identical twins is similar.
